# Surgical Treatment for Primary Lymphedema: A Systematic Review of the Literature

**DOI:** 10.1055/a-2253-9859

**Published:** 2024-04-08

**Authors:** Miguel Angel Gaxiola-García, Joseph M. Escandón, Oscar J. Manrique, Kristin A. Skinner, Beatriz Hatsue Kushida-Contreras

**Affiliations:** 1 Plastic and Reconstructive Surgery Department, Mexico's Children's Hospital (Hospital Infantil de México “Federico Gómez”), Mexico City, Mexico; 2 Division of Plastic and Reconstructive Surgery, Strong Memorial Hospital, University of Rochester Medical Center, Rochester, New York; 3 Department of Surgical Oncology, Strong Memorial Hospital, University of Rochester Medical Center, Rochester, New York; 4 Plastic and Reconstructive Surgery Department, Mexico's General Hospital (Hospital General de México), Mexico City, Mexico

**Keywords:** lymphedema, primary lymphedema, congenital lymphedema, lymphovenous anastomosis, lymph node transplant

## Abstract

This is a retrospective review of surgical management for primary lymphedema.

Data were extracted from 55 articles from PubMed MEDLINE, Web of Science, SCOPUS, and Cochrane Central Register of Controlled Trials between the database inception and December 2022 to evaluate the outcomes of lymphaticovenous anastomosis (LVA) and vascularized lymph node transfer (VLNT), and outcomes of soft tissue extirpative procedures such as suction-assisted lipectomy (SAL) and extensive soft tissue excision.

Data from 485 patients were compiled; these were treated with LVA ( *n* = 177), VLNT ( *n* = 82), SAL ( *n* = 102), and excisional procedures ( *n* = 124). Improvement of the lower extremity lymphedema index, the quality of life (QoL), and lymphedema symptoms were reported in most studies. LVA and VLNT led to symptomatic relief and improved QoL, reaching up to 90 and 61% average circumference reduction, respectively. Cellulitis reduction was reported in 25 and 40% of LVA and VLNT papers, respectively. The extirpative procedures, used mainly in patients with advanced disease, also led to clinical improvement from the volume reduction, as well as reduced incidence of cellulitis, although with poor cosmetic results; 87.5% of these reports recommended postoperative compression garments. The overall complication rates were 1% for LVA, 13% for VLNT, 11% for SAL, and 46% for extirpative procedures. Altogether, only one paper lacked some kind of improvement.

Primary lymphedema is amenable to surgical treatment; the currently performed procedures have effectively improved symptoms and QoL in this population. Complication rates are related to the invasiveness of the chosen procedure.

Lymphedema is a pathological entity characterized by volume enlargement of a body part caused by the accumulation of lymphatic fluid due to an affected lymphatic system; its causes are varied. When the blockage of lymphatic flow is due to surgery, trauma, radiation, or infection, the condition is termed secondary lymphedema; 1 in 1,000 people is affected. 1 Conversely, primary lymphedema entails a preexisting anomaly of the lymphatic system in patients with a family history or a genetic background for the disease. 2 The prevalence of primary lymphedema is 1.15 in 100,000 individuals 3 and involves either the lower extremity (91%) or upper extremity (9%). 2 4 5

Primary lymphedema has been classified into praecox to designate an early development of the disease, affecting mainly female patients aged from 10 to 24 years, and congenital, present at birth, and subdivided into simple and familial (Milroy's disease). 4 The term lymphedema *tarda* was subsequently introduced to designate the late presentation of the disease, which usually occurs after 35 years of age. 6

In the wide spectrum of congenital vascular malformations, primary lymphedema can appear as an isolated entity or be accompanied by other anomalies such as venous malformations or lymphangioma. 7 Also, primary lymphedema is an accompanying clinical feature of several syndromes with identified genetic associations: Hennekam syndrome (CCBE1), Noonan syndrome 1 (PTPN11), Emberger syndrome (GATA2), hypotrichosis-lymphedema-telangiectasia syndrome (SOX18), oculodentodigital dysplasia (GJA1), among others. 8 The usual clinical presentation in isolated primary lymphedema frequently shows an extremity with a woody, brawny texture, prominent veins, deep toe creases, “sky-jump” toenails, and papillomatosis (most severe over the second toe), and episodes of cellulitis and/or lymphangitis. 9

Various underlying pathological features have been identified in primary lymphedema, including hypoplasia, dilatation, and aplasia of the lymphatic trunks in 55, 24, and 14% of patients, respectively, 6 as well as diseased lymph nodes. 10 Magnetic resonance lymphangiography has confirmed defects of inguinal lymph nodes with mild or moderate dilatation of afferent lymph vessels in 17% of cases, lymphatic vascular anomalies (aplasia, hypoplasia, or hyperplasia) with no obvious defect of the draining lymph nodes in 32% of cases, and involvement of both lymph vessels and lymph nodes in 51% of cases. 11 These findings can potentially correlate to clinical features, considering the affected levels of the limb and the involvement of lymphatic hypoplasia. 11 12 It has been recognized that the defective development occurs in the later stage of lymphangiogenesis. 13 All these severe structural abnormalities have traditionally led primary lymphedema to be considered an incurable disease, unlike secondary lymphedema where originally the lymphatic structure and anatomy are normal, and continue to be until advanced stages, and the basic principle of surgical treatment is the restoration of flow in the severed lymphatic channels. 3

Hence, for the past 20 years, lymphaticovenous anastomosis (LVA) and its derivative mechanism through supermicrosurgery have become a popular physiological treatment modality for lymphedema 14 ; nevertheless, few studies have focused on the treatment of primary cases. 15 16 In consequence, nonsurgical treatment, compression therapy being the cornerstone, is critical in treating lymphedema, providing symptom relief, and halting the progression of the disease. 17 18 The results of these conservative therapies have been moderately successful: decreases in absolute limb volume (around 30%), decreases in body mass index, and improvement in quality of life (QoL) assessed through patient-reported outcome measures have been published. 19

Despite the above, several surgical treatment modalities are available nowadays. The vascularized lymph node transfer (VLNT) for primary lymphedema with hypoplastic lymph vessels has proven to be a beneficial physiological procedure 16 20 21 22 ; this modality works mainly in two ways: as a source for vascular endothelial growth factor, stimulating lymphangiogenesis in the affected limb, and drawing lymph forth into the venous circulation through a pressure gradient. 23 These fluid dynamics are further complicated by the role of the endothelial glycocalyx layer functioning as a monitor of fluid filtration from blood capillaries, causing most interstitial fluid to be reabsorbed by lymphatic rather than venous capillaries, as is now dictated by the revised Starling's principle. 24 25

Conversely, excisional and debulking procedures have been used as palliative surgeries for lymphedema. These include the Charles procedure, which is performed predominantly for advanced stages of lymphedema, resulting in evident scarring with tissue breakdown and poor cosmetic results, as well as lymphorrhea, recurrence, and residual distal edema 26 27 ; and suction-assisted lipectomy (SAL), which started as a conjunct procedure for compression-resistant lymphedema. 28 29

Although lymphedema has been an object of special attention in recent years, the special considerations of primary lymphedema etiopathology, concurrently with the unavoidable long-standing progression of the disease before an accurate diagnosis is made, have altogether contributed to the current lack of well-established protocols in the surgical treatment for this condition. Indeed, primary lymphedema is considered a rare or orphan disease. 30 Therefore, in this study, we aimed to perform a systematic review of the literature focusing on the reported outcomes of surgical treatment in the context of primary lymphedema of the extremities.

## Methods

### Protocol and Search Strategy

This review was performed commensurate with the Preferred Reporting Items for Systematic Reviews and Meta-Analyses (PRISMA) guidelines (PRISMA Checklist available online). 31 32 A comprehensive search design by author J.M.E. across PubMed MEDLINE, Web of Science, SCOPUS, and Cochrane Central Register of Controlled Trials was performed from database inception through December 2022. The terms “Lymphedema,” “Primary,” “Hereditary,” “Congenital,” “Praecox,” “Tarda,” “Meige's syndrome,” “Milroy's disease,” “Lymph node transfer,” “Lymphovenous anastomosis,” “Liposuction,” “Lipectomy,” “lymph node transplant,” “Excision,” and “radical reduction preservation perforators” were used as keywords with Boolean operators in several combinations (see Supplementary Table S1 [available in the online version only], which exhibits the specific search terms used for the different databases).

### Inclusion and Exclusion Criteria

We included original articles written in English, reporting outcomes and surgical techniques for the management of primary lymphedema of extremities in human patients. Preclinical studies and survey studies were excluded. Studies reporting outcomes where multiple patients with primary and secondary lymphedema were included when the outcomes of primary lymphedema were explicitly distinguished from the analysis. Otherwise, studies dealing with primary and secondary lymphedema where data were aggregated without distinction were excluded. Studies reporting outcomes of the surgical management of exclusively lymphatic malformations, malignancies secondary to lymphedema, or genital lymphedema, were excluded.

### Study Selection and Data Extraction

Once duplicated citations were excluded, two independent authors (B.H.K-C. and J.M.E) evaluated the included references based on the title and abstract. Subsequently, a full-text assessment was accomplished in the remaining studies. Disagreements through this two-step process were solved by a third author (M.A.G-G.). Two authors performed data extraction independently. Extracted data included author and year, location, number of patients, age, lymphedema stage, duration of lymphedema, associated syndromes or comorbidities, surgical technique, adjuvant procedures, postoperative protocol, outcomes, complications, and follow-up. Cumulative estimates were calculated as weighted means.

### Quality Assessment and Risk of Bias

Appraisal of the levels of evidence was performed independently by two reviewers (J.M.E. and M.A.G-G.) using the Oxford Centre for Evidence-Based Medicine (OCEBM) ( Supplementary Table S2 [available in the online version only]). 33 The risk of bias was evaluated by operating the Newcastle–Ottawa Scale (NOS; Supplementary Table S3 [available in the online version only]) for observational cohort studies, and the Methodological Quality Assessment Tool (MQAT) for case reports and case series ( Supplementary Table S4 [available in the online version only]). 34 35

## Results

### Literature Search

**Fig. 1:**
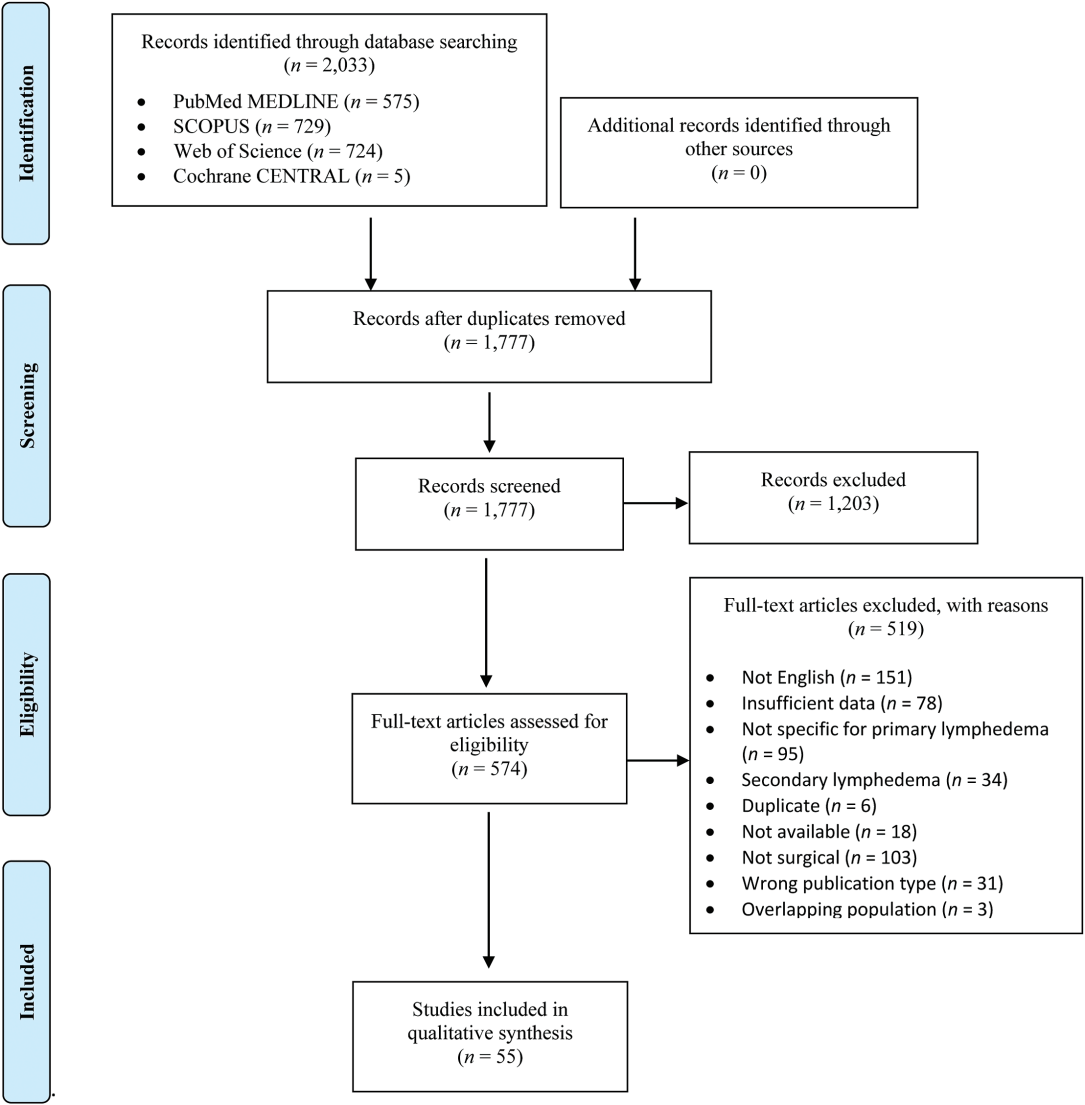
Preferred Reporting Items for Systematic Reviews and Meta-Analyses flow chart. CENTRAL, Central Register of Controlled Trials.

Overall, 2,033 citations were identified during the electronic bibliographic search. After duplicated references were eliminated, 1,777 records were screened, and 1,203 were excluded based on the title and abstract review. Following a full-text review, 55 articles met the inclusion criteria and were selected for data extraction. The PRISMA flow chart can be seen in Fig. 1 . 5 21 22 26 36 37 38 39 40 41 42 43 44 45 46 47 48 49 50 51 52 53 54 55 56 57 58 59 60 61 62 63 64 65 66 67 68 69 70 71 72 73 74 75 76 77 78 79 80 81 82 83 84

An overview of the studies' characteristics is displayed in Table 1 .

**Table 1:** Overview and quality assessment of included studies reporting surgical outcomes of primary lymphedema

Author, year	Journal	Location	OCEBM	NOS	Patients ( *n* )	Age (years)	Site	Grading	Lymphedema duration (years)	Syndrome or comorbidities	Follow-up (months)
MacKmull et al, 1950	Plastic and Reconstructive Surgery	Philadelphia, Pennsylvania	4	5 a	1	25	LE	NR	25	NR	8
Fonkalsrud et al, 1969	Journal of Pediatric Surgery	Los Angeles, California	4	3 a	4	(Range, 3–15)	LE	NR	NR	NR	>6
Tilley et al, 1974	The Canadian Medical Association Journal	Toronto, Canada	4	4 a	1	40	LE	III ISL	26	NR	10
Dellon et al, 1977	Plastic and Reconstructive Surgery	Baltimore, Maryland	4	4 a	9	31 (range, 22–40)	LE	NR	Range, 12–18	NR	127 (range, 14–277)
					1	1.5	UE	NR	1.45	NR	216
Feins et al, 1977	Journal of Pediatric Surgery	Boston, Massachusetts	4	5	38	Range, 1–19	LE ( *n* = 36) UE ( *n* = 2)	NR	NR	NR	Range, 1–60
Smeltzer et al, 1985	Pediatrics	Rochester, Minnesota	4	5	16	NR	NR	NR	NR	Milroy's disease ( *n* = 1) Meige's disease ( *n* = 3)	Range, 0–324
Louton et al, 1989	Annals of Plastic Surgery,	Charleston, South Carolina	4	3 a	1	26	LE	NR	13	NR	NR
Mavili et al, 1994	Lymphology	Ankara, Turkey	4	4 a	4	NR	LE	NR	NR	NR	Range, 12–36
Dumanian et al, 1996	Lymphology	Pittsburgh, Pennsylvania	4	4 a	1	35	LE	NR	15	NR	180
Koshima et al, 2003	Journal of Reconstructive Microsurgery	Okayama, Japan	4	4 a	4	33 (range, 12–53)	LE	NR	9.25 (range, 2–24)	NR	93 (range, 60–108)
Fraga et al, 2004	Lymphology	São Paulo, Brazil	4	4 a	1	21	UE	NR	15	NR	0.5
Hosnuter et al, 2006	Medical Science Monitor	Zonguldak, Turkey	4	4 a	1	47	LE	III ISL	16	NR	12
Greene et al, 2006	Plastic and Reconstructive Surgery	Boston, Massachusetts	4	4 a	1	34	LE	NR	10	Spina bifida Paraplegia Hydrocephalus Ventriculoperitoneal shunt	18
Espinosa et al, 2009	Journal of Vascular Surgery	Mexico City, Mexico	4	4 a	1	26	LE	III ISL	10	NR	14
Eryilmaz et al, 2009	Aesthetic Plastic Surgery	Ankara, Turkey	4	5 a	1	29	LE	NR	20	NR	22
van der Walt et al, 2009	Annals of Plastic Surgery	Cape Town, South Africa	4	5	8	34.8 (range, 13–57)	LE	NR	17.6 (range, 12–31)	NR	27.3 (range, 12–90)
Karonidis et al, 2010	Annals of Plastic Surgery	Kaohsiung, Taiwan	4	6	8	21.6 (range, 16–51)	LE	Advanced	6.37 (range, 3–10)	NR	36
Pereira et al, 2010	International Journal of General Medicine	São José do Rio Preto, Brazil	4	3 a	2	59 (range, 65–64)	LE	III ISL	NR	NR	3
Mihara et al, 2011	Clinical Radiology	Tokyo, Japan	4	4 a	2	52	LE	II ISL	NR	NR	23.6
Yamamoto et al, 2011	Journal of Plastic, Reconstructive and Aesthetic Surgery	Tokyo, Japan	4	4 a	2	20 (range, 15–25)	LE and scrotum	NR	20 (range, 15–25)	NR	34 (range, 15–53)
Auba et al, 2012	Microsurgery	Pamplona, Spain	4	4 a	1	52	LE	III Campisi	24	NR	18
Suehiro et al, 2012	Surgery Today	Yamaguchi, Japan	4	4 a	1	25	LE and scrotum	NR	12	Absence of the thoracic duct and dilated iliac lymph trunks	12
Yamamoto et al, 2013	PLoS ONE	Tokyo, Japan	4	6	6	Range, 25–71	LE	I II III ISL	Range, 0.75–18	NR	6
Ayestaray et al, 2014	Journal of Reconstructive Microsurgery	Evry, France	4	5 a	1	34	LE	NR	23	Turner syndrome	12
Gómez Martín et al, 2014	Journal of Plastic, Reconstructive and Aesthetic Surgery	Madrid, Spain	4	4 a	1	57	LE	NR	5	NR	5
Qiu et al, 2014	Plastic and Reconstructive Surgery - Global Open	Taoyuan, Taiwan	4	4 a	1	13	LE	NR	NR	Klippel–Trenaunay	3
Akita et al, 2015	Annals of Plastic Surgery	Chiba, Japan	4	6	1	34	LE	NR	13	NR	12
Hara et al, 2015	Plastic and Reconstructive Surgery	Tokyo, Japan	4	6	62	42 (range, 10–90)	LE	1 ( *n* = 8) 2a ( *n* = 23) 2b ( *n* = 46) 3 ( *n* = 2) ISL	10.6 (range, 0.1–52)	NR	19.5 (range, 5.6–54.3)
Koshima et al, 2015	Journal of Reconstructive Microsurgery	Tokyo, Japan	4	5 a	2	17.5 (range, 15–20)	LE	NR	2.25 (range, 2–2.5)	NR	3.5 (range, 3–4)
Chen et al, 2015	Journal of Reconstructive Microsurgery	Iowa City, Iowa	4	5 a	1	50	LE	IV Campisi	NR	NR	Range, 6–9
Ito et al, 2016	Microsurgery	Taoyuan, Taiwan	4	5 a	2	32.5 (range, 29–36)	LE	1.5 Cheng's	8 (range, 2–14)	NR	10.5 (range, 3–19)
Gennaro et al, 2016	European Review for Medical and Pharmacological Sciences	Siena, Italy	4	6	8	42 (range, 16–56)	LE	I ( *n* = 1) II ( *n* = 6) III ( *n* = 1) ISL	7.85 (range, 2–15)	NR	36
					1	48	UE	III ISL	4	NR	36
Greene et al, 2016	Annals of Plastic Surgery	Boston, Massachusetts	4	4 a	8	41.87 (range, 17–66)	LE	NR	NR	NR	36
Lee et al, 2016	Lymphology	Los Angeles, California	4	5 a	1	65	LE	NR	35	NR	15
Yamamoto et al, 2016	Journal of Plastic Reconstructive and Aesthetic Surgery	Tokyo, Japan	4	5 a	1	49	LE	NR	5	NR	18
Chen et al, 2016	Journal of Reconstructive Microsurgery	Iowa City, Iowa	4	6	4	54.5 (range, 50–62)	LE	III ( *n* = 1) IV ( *n* = 3) Campisi	NR	NR	12
Mihara et al, 2016	Plastic and Reconstructive Surgery	Saitama, Tokyo	4	5	15	Range, 24–94	LE	I–III ISL	NR	NR	Range, 6–51
Lamprou et al, 2017	British Journal of Surgery	Drachten, The Netherlands	4	6	47	43.6 (range, 12–4)	LE	“End stage”	20 (range, 10–33)	NR	12
Lee et al, 2017	Microsurgery	Seoul, South Korea	4	5 a	7	37 (range, 11–58)	LE	II ( *n* = 4) III ( *n* = 3) Campisi	6.78 (range, 1–15)	NR	24
Stewart et al, 2018	Journal of Plastic Reconstructive and Aesthetic Surgery	Dundee, United Kingdom	4	6	42	41 (range, 20–68)	LE	II–III ISL	20 (range, 4–45)	NR	16 (range, 6–48)
Borz et al, 2018	Annali Italiani di Chirurgia	Munes, Romania	4	4 a	18	18	LE and scrotum	NR	14	Praecox	3
Cheng et al, 2018	Plastic and Reconstructive Surgery - Global Open	Taoyuan, Taiwan	4	6	17	31.5 (range, 2–57)	LE	I ( *n* = 2) II ( *n* = 10) III ( *n* = 2) IV ( *n* = 5) Cheng's	4.51 (range, 0.25–9.6)	Klippel–Trenaunay ( *n* = 4)	18.2 ± 8.9
Sachanandani et al, 2018	Journal of Surgical Oncology	Taoyuan, Taiwan	4	5 a	3	25 (range, 13–43)	LE	I ( *n* = 1) IV ( *n* = 4) Cheng's	13 (range, 8–18)	Klippel–Trenaunay ( *n* = 2) Concomitant vascular lesions ( *n* = 3)	23 (range, 19–30)
Giacalone et al, 2019	Journal of Clinical Medicine	Mechelen, Belgium	4	4 a	1	27	LE	NR	27	Complex lymphatic malformation	4
Maruccia et al, 2019	Microsurgery	Bari, Italy	4	5 a	1	32	LE	III ISL	3	NR	3
AlJindan et al, 2019	Plastic and Reconstructive Surgery	Taoyuan, Taiwan	4	6	15	NR	LE ( *n* = 14) UE ( *n* = 1)	1.2 Cheng's	NR	NR	14.2 (range, 12.3–16.1)
Bolleta et al, 2020	Journal of Surgical Oncology	Taichung, Taiwan	4	5	15	16 ± 0.8	LE	II–III Cheng's	16 ± 0.8	Milroy's disease	20.2 ± 2.8
Robertson et al, 2020	Journal of Vascular Surgery	Cincinnati, Ohio	4	4 a	2	42.5 (range, 35–50)	LE	NR	4.5 (range, 3–6)	NR	12
Damstra et al, 2020	Journal of Clinical Medicine	Drachten, The Netherlands	4	6	28	44.7 (range, 32–66)	LE	III ISL	27.5 (range, 6–36)	NR	54 (range, 36–60)
Ciudad et al, 2020	Microsurgery	Taichung, Taiwan	4	6	11	(range, 26–53)	LE and UE	II and III ISL	3.5 (range, 0.6–6.3)	NR	32.8 (range, 24–49)
Cheng et al, 2020	Microsurgery	Taoyuan, Taiwan	4	5 a	9	9.2 (range, 2–19)	LE	2.6 ± 1.6 Cheng's	9.3 (range, 2–19)	NR	38.4 (range, 16–63)
Drobot et al, 2021	Journal of Vascular Surgery	Hiroshima, Japan	4	5	22	34	LE	II ISL	7.3	NR	9 (range, 3–24)
Onoda et al, 2021	Journal of Vascular Surgery	Kagawa, Japan	4	5	2	46 (range, 30–62)	LE	II ISL	NR	NR	31 (range, 6–48)
Scaglioni et al, 2021	Microsurgery	Lucerne, Switzerland	4	5	1	46	LE	III Campisi	NR	NR	9
					2	4.5 (range, 2–7)	UE	2.5 Cheng's	4 (range, 3–5)	NR	37 (range, 31-43)
Hayashi et al, 2022	Journal of Clinical Medicine	Chiba, Japan	4	5	26	44.2 (range, 16–82)	LE	1 ( *n* = 3) 2a ( *n* = 15) 2b ( *n* = 14) 3 ( *n* = 1) ISL	8.6 (0.8–29)	NR	17.5 (range, 6–36)

Abbreviations: ISL, International Society of Lymphology; LE, lower extremity; OCEBM, Oxford Centre for Evidence-Based Medicine: Levels of Evidence; NOS, Newcastle–Ottawa Scale; NR, not reported; UE, upper extremity.

a Case reports and case series in which the Methodological Quality Assessment Tool proposed by Murad et al 34 was used.

### Quality Assessment

All studies had a level of evidence of 4 using the OCEBM instrument ( Table 1 ), indicating that most studies included were case series and poor-quality cohort and case–control studies. Most case series and case reports had a moderate risk of bias when using the MQAT as 12 studies scored 5, 19 scored 4, and 3 scored 3. The evaluation of the methodological quality of cohort studies was as follows: 12 studies had an NOS score of 6, and 9 scored 5, which showed a low-to-moderate risk of bias.

### Demographic and Clinical Characteristics

This review included 485 patients with primary lymphedema. The average age was 36.44 years and ranged from 1 to 94 years, reported in 52 studies. Seven (12%) and 53 (96%) articles reported the surgical management for upper extremity lymphedema and lower extremity lymphedema, respectively. The average follow-up was 24.74 months (range, 1–324 months), reported in 47 studies. The average duration of lymphedema before the surgical intervention reported in the articles was 14.2 years (range, 1 month–52 years), reported in 365 patients. Different lymphedema staging systems were reported in the included studies; the most common was the International Society of Lymphology (ISL) scale ( *n* = 17), followed by the Cheng's lymphedema grading scale ( *n* = 7) and the Campisi staging system ( *n* = 5). See Table 1 .

Several congenital malformations and syndromes were associated with primary lymphedema including Milroy's disease ( *n* = 16), Klippel–Trenaunay syndrome ( *n* = 7), Meige's disease ( *n* = 3), Turner syndrome ( *n* = 1), spina bifida with hydrocephalus ( *n* = 1), absence of the thoracic duct ( *n* = 1), congenital vascular lesions ( *n* = 3), and complex lymphatic malformations ( *n* = 1).

### Lymphaticovenous Anastomosis

This procedure has been reported since 2003. Twenty-four studies adequately reported the surgical outcomes of 177 patients with primary lymphedema treated with LVAs. Most studies reported LE (91%) surgical outcomes, and only two reported outcomes of the UE (8%). Staging of lymphedema was heterogeneously reported among studies. The most common stages treated with LVAs were ISL II ( *n* = 130) and ISL I ( *n* = 13). Only seven patients with lymphedema stage III were treated using this modality. When using Cheng's classification, most patients were in stage II to III (n = 58). When using the Campisi staging system, most patients were in stage II ( *n* = 4), followed by stage III ( *n* = 3) and IV ( *n* = 1).

The average number of LVAs per patient was 3.44 (range, 1–9), reported in 174 patients. The most common LVA techniques were the end-to-side, end-to-end, or side-to-end technique; nonetheless, several studies reported the use of π-shaped LVAs, octopus LVAs, and side-to-end anastomosis through temporary lymphatic expansion. An overview of the results is displayed in Table 2 . Surgical outcomes were not homogeneously reported. In most studies, an improvement of the LE lymphedema index, the QoL, and lymphedema symptoms, as well as a reduction of the cross-sectional area, episodes of cellulitis, the need for compression garments, and circumferential measures were reported. Some papers reported marginal improvements, for example, Mihara et al reported an average reduction rate of 2.7% in limb circumference, 69 while the same author had previously reported average size reductions of around 90%. 51 In contrast, Auba et al reported an increment in the limb perimeter in comparison to preoperative measures. 53 Hara et al also reported that the LE circumference increased following LVA treatment in patients with an onset age of <11 years; but significantly decreased in patients with an onset age of >11 years. 15 QoL improvement was represented by diminution or absence of cellulitis episodes with less need for compression garments 77 ; reported explicitly in at least 25% of papers. Systematic assessment of the QoL was seldom reported using the Lymphoedema Quality of Life Questionnaire (LYMQoL). 16 The overall complication rate was 1%. The most common complications reported were several episodes of a lymphatic fluid leak in one patient and failure of the anastomosis. 52 55

**Table 2:** Studies reporting surgical outcomes of primary lymphedema using lymphaticovenous anastomosis

Author, year	Patients ( *n* )	Site	Surgical technique	Other procedures	Postoperative treatment	Outcomes	Complications
Koshima et al, 2003	4	LE	LVA Number of anastomoses (mean): 4.25 (range, 2–5)	Fat flap	Compression garments	Remarkable reduction in the circumference (8 cm each in the B/L lower legs) Patients achieved a 55.6% reduction of the excess circumference	NR
Mihara et al, 2011	2	LE	LVA Number of anastomoses (mean): 3.5 (range, 3–4)	NR	NR	The average size reduction was 90.15% Degree of limb hardness decreased from 2 to 1	NR
Yamamoto et al, 2011	2	LE and scrotum	Multisite LVA Number of anastomoses (mean): 6 (range, 3–9)	NR	NR	No recurrence ( *n* = 2)	Several episodes of lymphorrhea ( *n* = 1)
Auba et al, 2012	1	LE	LVA	NR	Limb elevation	The average preoperative limb perimeter increased from 32.1 to 32.9 cm	–
Suehiro et al, 2012	1	LE and scrotum	LVA ( *n* = 2)	NR	Medium-chain triglycerides supplement Compression therapy	2,000-mL reduction from the initial presentation Episodes of cellulitis decreased from every month to none	NR
Yamamoto et al, 2013	6	LE	SEATTLE ( *n* = 2) Standard LVA ( *n* = 4)	NR	NR	The LEL index decreased 18.2 ± 15.9 in patients with primary lymphedema LEL index reduction in SEATTLE group was significantly greater that in non-SEATTLE group	11% of LVAs resulted in anastomosis failure
Bekara et al, 2014	1	LE	LVA π-shaped Number of anastomoses: 4	NR	NR	The circumferential reduction rate was 17% Cross-sectional area reduction rate was 32.2% Average volume reduction rate was 36.5%	No complications
Akita et al, 2015	1	LE	Multiple LVA	NR	NR	LEL index improved from 258.8 to 245.2 for the right leg, and from 292.5 to 265.5 for the left leg	NR
Hara et al, 2015	62	LE	LVA ( *n* = 79) Number of anastomoses (mean): 4.5 (range, 0–9)	NR	NR	LE circumference increased after LVA in patients with an onset age of 1 year or later and before age 11 years, but significantly decreased in patients with an onset age older than 11 years	NR
Ito et al, 2015	2	LE	LVA Number of anastomoses (mean): 2	NR	Compression therapy	The mean circumference reduction rate was 70.4%	NR
Yamamoto et al, 2015	1	LE	Number of drainage pathways/octopus LVA: 14 in 4	NR	NR	Postoperative Campisi stage: II Reduction of the LEL index from 378 to 352	NR
Gennaro et al, 2016	8	LE	LVA Number of anastomoses (mean): 5.75 (range, 5–7)	NR	Lymphatic drainage and compression stocking	Average size reduction was 61% (range 41–87%)	No complications
	1	UE	LVA Number of anastomoses: 5	NR	Lymphatic drainage and compression stocking	41% size reduction	No complications
Yamamoto et al, 2016	1	LE	LT-VLNT + ELLA LVA Number of anastomoses: 2	NR	Compression garment	No episode of cellulitis with reduced degree of compression treatment Lymphedematous volume decreased from 306 to 264 in terms of LEL index	No complications
Chen et al, 2016	4	LE	LVA Number of anastomoses (mean): not specified	NR	NR	12-month postoperative Campisi stage II ( *n* = 2) and III ( *n* = 2) Significant improvement in QoL scores: decreased 10.5 Overall reduction of 17 point in the LEL index	NR
Mihara et al, 2016	15	LE	Multisite LVA	NR	NR	The average reduction rate was 2.7%	NR
Lee et al, 2017	7	LE	LVA Number of anastomoses (mean): 2.42 (range, 1–3)	NR	Physical therapy	Reduction rate of volume: 39.2 ± 43.9 at 6 months, 20.2 ± 44.2 at 12 months, 38.7 ± 57.4 at 24 months	NR
Cheng et al, 2018	17	LE	LVA ( *n* = 4) Number of anastomoses: 1	SM-VLNT ( *n* = 15)	NR	Following LVA: Limbs had a mean 1.9 ± 2.9 cm circumference reduction Reduction in body weight 6.6 ± 5.9 kg in VLNT and of 1.7 ± 0.6 kg in LVA LYMQoL improvement for LVA	NR
Giacalone et al, 2019	1	LE	LVA	NR	NR	The difference in volume between the left and right leg was reduced from 1,222 to 224 mL	No complications
AlJindan et al, 2019	15	LE ( *n* = 14) UE ( *n* = 1)	LVA Number of anastomoses (mean): 1	NR	NR	Episodes of cellulitis were significantly reduced from 1.7 times/year to 0.7 times/year Circumferential Difference improvement was 3% Patients did not need compression garments postoperatively	No complications
Drobot et al, 2020	22	LE	LVA Number of anastomoses (mean): 3.1 (range, 1–4)	NR	Compression therapy protocol (3 months)	Absolute volume change (in milliliters) at 6 months postoperatively: 372 ± 52 (55%)	No complications
Cheng et al, 2020	2	UE and LE	LVA	NR	None of the patients used compression garments postoperatively	The mean limb circumferential difference was improved by 5.5% (preoperative, 7.7; postoperative 5.5) Episodes of cellulitis decreased by 2.2 times/year	No complications
Onoda et al, 2020	2	LE	LVA Number of anastomoses (mean): 4.5 (range, 4–5)	NR	Inpatient complex decongestive physiotherapy	Percentage reduction from admission to follow-up: 19.4% (range, 8.1–30.7%)	No complications
Scaglioni et al, 2020	1	LE	LVA Number of anastomoses (mean): 1 deep LVA and 5 superficial LVAs	NR	NR	Initial Campisi stage III to Final Campisi stage Ib Overall improvement of symptoms	NR
Hayashi et al, 2022	26	LE	LVA Number of anastomoses (mean): 8.7 total; posterior side 3.5 LVAs and medial–anterior side 4.6 LVAs	Previous LVAs	NR	Mean reduction of the LEL index 5.3–32.9 (18.1) After second procedure: 10.5 ± 4.5 in posterior side LVAs, 5.5 ± 3.6 in medial–anterior side LVAs	NR

Abbreviations: B/L, bilateral; ELLA, efferent lymphaticolymphatic anastomosis; LVA, lymphaticovenous anastomosis; LE, Lower extremity; LEL, lower extremity lymphedema; LYMQoL, Lymphoedema Quality of Life Study; NR, not reported; SEATTLE, side-to-end anastomosis through temporary lymphatic expansion; SM-VLNT, submental-vascularized lymph node transfer; UE, upper extremity; VLNT, vascularized lymph node transfer.

### Vascularized Lymph Node Transfer

We found 12 articles reporting outcomes of VLNT for primary lymphedema, accounting for 82 treated patients. An overview of the results is displayed in Table 3 . This technique was used mainly for the treatment of LE lymphedema. Pedicled VLNTs were described in two series. Fonkalsrud et al 37 reported an omentum transposition as described by Goldsmith 37 , while Borz et al reported modified enteromesenteric bridging. 72 The remaining eight studies reported the use of free VLNT, including the submental-VLNT (SM-VLNT; 33.33%), groin-VLNT (8.3%), vascularized omental lymph node transfer (8.3%), gastroepiploic-VLNT (16.6%), lateral thoracic-VLNT (16.6%), and the first web space-VLNT (8.3%).

**Table 3:** Studies reporting surgical outcomes of primary lymphedema using vascularized lymph node transfer

Author, year	Patients ( *n* )	Site	Surgical technique	Other procedures	Postoperative treatment	Outcomes	Complications
Fonkalsrud et al, 1969	1	LE	Omentum transposition as described by Goldsmith	NR	NR	Leg swelling subsided during the first 6 months after operation, but gradually returned as the patient became overweight	NR
Gómez Martín et al, 2014	1	LE	G-VLNT (First stage) LT-VLNT (Second stage)	NR	Manual drainage, compressive bandages	Average circumference reduction rate of 59.4% No episodes of cellulitis	No complications
Qiu et al, 2014	1	LE	SM-VLNT	NR	NR	Symptomatic improvement Circumferential reduction rates in the right LE at 15 cm AK, 15 cm BK, and 10 cm AA were 50, 53.3, and 33%, respectively	No complications
Koshima et al, 2015	2	LE	FWS-VLNT ( *n* = 2)	NR	Compression therapy ( *n* = 1)	Dramatic improvement without any postoperative complications	NR
Yamamoto et al, 2016	1	LE	LT-VLNT + ELLA	LVA	Compression garment	No episode of cellulitis with reduced degree of compression treatment, and lymphedematous volume decreased from 306 to 264 in terms of lower extremity lymphedema index were reported	No complications
Borz et al, 2018	18	LE and scrotum	Modified enteromesenteric bridging	NR	NR	Decrease of the mid-calf diameters with 5.2 cm on the right and 4.8 cm on the left	No complications
Cheng et al, 2018	17	LE	SM-VLNT ( *n* = 15)	LVA ( *n* = 4)	NR	Limbs that underwent VLNT had a mean 3.7 ± 2.9 cm circumference reduction Reduction in body weight 6.6 ± 5.9 kg in VLNT and of 1.7 ± 0.6 kg in LVA LYMQoL in overall score improvement for VLNT and LVA	NR
Sachanandani et al, 2018	3	LE	SM-VLNT ( *n* = 3)	LVA ( *n* = 1)	NR	Final circumferential reduction rate of 39.16% above the knee and 34.5% below the knee	Hematoma ( *n* = 1) Venous thrombosis ( *n* = 2) Revision surgery ( *n* = 2)
Bolleta et al, 2019	15	LE	GE-VLNT ( *n* = 15)	Brorson's secondary SAL	NR	The average circumference reduction was of 5.9 ± 1.2 cm at mid-thigh, 4.9 ± 2.2 cm at mid-calf, 3.7 ± 0.8 cm at the ankle, and 1.7 ± 0.9 cm at mid-foot Tonicity overall was reduced by 6.8 ± 0.8% No episodes of cellulitis	No complications
Maruccia et al, 2019	1	LE	GE-VLNT—Laparoscopic	CDP—1 week preoperatively	Compression garments	The limb circumference reduction was 62.5% below the knee, and 41.4% above the knee	No complications
Ciudad et al, 2020	11	LE and UE	G-VLNT SC-VLNT GE-VLNT—Open and Laparoscopic A-VLNT IC-VLNT	NR	NR	Circumference reduction rate, % (mean ± SD): 18.9 ± 14.0 The positive circumference reduction was not significantly associated with VLNT	NR
Cheng et al, 2020	9	LE	SM-VLNT ( *n* = 9) Volt ( *n* = 1)	NR	NR	The mean limb circumferential difference was improved by 17.2% (preoperative, 26.98; postoperative 22.34) Episodes of cellulitis decreased by 2.67 times/year No use of compression garments postoperatively	Venous congestion with successful salvage (n = 3) Partial skin paddle necrosis (n = 2)
	2	UE	SM-LNT ( *n* = 1)	NR	NR	The mean limb circumferential difference was improved by 61% (preoperative, 22.7; postoperative, 8.3) Episodes of cellulitis decreased by 3 times/year	No complications

Abbreviations: AA, above the ankle; AK, above the knee; BK, below the knee; A-VLNT, appendicular VLNT; CDP, complex decongestive physiotherapy; ELLA, efferent lymphaticolymphatic anastomosis; FWS-VLNT, first web space VLNT; G-VLNT, groin VLNT; GE-VLNT, gastroepiploic VLNT; LE, lower extremity; IC-VLNT, ileocecal VLNT; LT-VLNT, lateral thoracic; NR, not reported; VLNT; LVA, lymphaticovenous anastomosis; LYMQoL, Lymphoedema Quality of Life Questionnaire; SAL, suction-assisted lipectomy; SC -VLNT, supraclavicular VLNT; SD, standard deviation; SM-VLNT, submental-VLNT; UE, upper extremity; VLNT, vascularized lymph node transfer; VOLNT, vascularized omental lymph node transfer.

a Although labeled differently, these flaps correspond to the same procedure.

The outcomes were not reported uniformly; however, some reports stated that the average circumference reduction rate ranged from 17.2 to 61%, tonicity was reduced by 6.8 ± 0.8%, and the episodes of cellulitis decreased by 2.67 to 3 times/year during a follow-up ranging from 16 to 63 months. As a whole, a reduction in cellulitis episodes was reported explicitly in at least 40% of papers. Qualitatively, most studies reported improved symptoms and QoL. 21 22 57 58 73 74 76 Unsatisfactory results were reported in the patient managed with omentum transposition: the leg swelling initially subsided during the first 6 months postoperatively, but the edema gradually returned as the patient became overweight. The overall complication rate was 13%; these included hematoma formation ( *n* = 1), venous congestion or thrombosis ( *n* = 4), and microsurgical revisions ( *n* = 4). 22 73

### Suction-assisted Lipectomy

One hundred and two patients were treated in 8 studies reporting the use of SAL; among them, one specifically used a two-staged SAL technique. An overview of the results is shown in Table 4 . Most of the patients had stage II to III ISL lymphedema or had “end-stage” lymphedema. The mean reduction of original excess volume ranged from 71.9 to 94%. 64 71 Qualitatively, several articles reported a reduction in cellulitis episodes and an improvement of the QoL. 40 46 64 Remarkably, 87.5% of studies highlighted the importance of postoperative compression bandages. The overall complication rate was 11%; these included limited liposuction in certain areas ( *n* = 1), skin necrosis ( *n* = 5), significant blood loss ( *n* = 4), cellulitis ( *n* = 1), the requirement of further procedures ( *n* = 1), decubitus ulcers ( *n* = 1), and temporary peroneal nerve palsy ( *n* = 2). 64 65 71

**Table 4:** Studies reporting surgical outcomes of primary lymphedema using suction-assisted lipectomy and excisional procedures

Author, year	Patients ( *n* )	Site	Surgical technique	Other procedures	Postoperative treatment	Outcomes	Complications
**Mainly suction-assisted lipectomy**							
Louton et al, 1989	1	LE	SAL	NR	Excision of redundant tissue, 4 days postoperatively	Large amount of redundant skin and subcutaneous tissue draped over an otherwise normal leg	The fibrotic areas over the dorsum of the feet were difficult to debulk
Greene et al, 2006	1	LE	SAL	NR	Pressure bandaging	Lower extremity circumferential measurements corresponded to a 75% reduction from her preoperative volume	NR
Espinosa et al, 2009	1	LE	SAL	NR	40 mm Hg compression bandages	Volume of the legs decreased from 10.7 L and 8.9 L to 6.4 L and 6.1 L, postoperatively Cellulitis has not occurred, and antibiotics have not been required so far	No complications
Eryilmaz et al, 2009	1	LE	Two-stage SAL	NR	NR	20% reduction from his first preoperative measurements	No complications
Greene et al, 2016	8	LE	SAL	NR	Compression bandages	The mean reduction in excess extremity volume was 73% (range, 48–94%) Better quality of life; none exhibited recurrence	Skin necrosis ( *n* = 2) Significant blood loss ( *n* = 2) Cellulitis ( *n* = 1) Surgical debridement ( *n* = 1)
Lamprou et al, 2016	47	LE	SAL	NR	Compression bandages	Average size reduction was 79% and absolute volume reduction of 3,670 mL compared with preoperative affected leg volume A reduction from 8 attacks of cellulitis to 0.2 attacks per year	Decubitus ulcer ( *n* = 1)
Lee et al, 2016	1	LE	SAL	NR	Continuous compression garment	A stable overall excess volume reduction of 4,227 mL (86%) was achieved at 15 months postoperatively which remained stable thereafter	NR
Stewart et al, 2017	42	LE	SAL	NR	Wrap garments	71.9% reduction of original excess volume at 3 months postoperative 84.3% reduction of original excess volume at 1 year postoperative	Skin necrosis ( *n* = 3) Temporary peroneal nerve palsy ( *n* = 2) Significant blood loss ( *n* = 2)
**Mainly excisional procedures**							
MacKmull et al, 1950	1	LE	Two-stage modified Kondoleon–Sistrunk Procedure	NR	Elevation 75 degrees	Remarkable reduction in size of the leg No recurrence of lymphangitis	Internal saphenous nerve injury ( *n* = 1)
Fonkalsrud et al, 1969	3	LE	Skin-sparing subcutaneous tissue excision	NR	Elastic bandages	Adequate cosmesis during postoperative assessment	Transfusion of blood units ( *n* = multiple) Delayed wound healing ( *n* = 2)
Tilley et al, 1974	1	LE	Charles procedure—STSG Staged-tissue excision	NR	NR	Marked improvement in function; the appearance is less than ideal but is vastly improved	Transfusion of blood units ( *n* = 2) Dermatosis ( *n* = 1) Skin graft loss ( *n* = 1)
Dellon et al, 1977	9	LE	Charles procedure	NR	Wrap garments	Excellent functional and cosmetic outcomes Lymphedema in the dorsum of the foot ( *n* = 2)	Crevices and pits ( *n* = 1) Chronic ulceration ( *n* = 1) Scar revision and release ( *n* = 1)
	1	UE	Charles procedure—FTSG	NR	NR	Excellent functional and cosmetic outcomes	Scar revision and release ( *n* = 1)
Feins et al, 1977	38	LE (n = 36) UE (n = 2)	Single-stage modified Homan's procedure ( *n* = 26) Double-stage ( *n* = 10) Triple-stage ( *n* = 2)	NR	Compression therapy 3 months	Improvement of symptoms ( *n* = 38) No episodes of lymphangitis and cellulitis	Wound dehiscence ( *n* = 2) Revision surgery ( *n* = 1) Seroma ( *n* = 1)
Smeltzer et al, 1985	16	NR	Homan's procedure ( *n* = 7) Charles procedure ( *n* = 3) Genital procedure ( *n* = 4)	Thompson buried flap ( *n* = 7)	NR	Scores: (excellent, good, fair, or poor): - Homan's procedure (fair: 3; poor: 4) - Charles procedure (good: 1; fair: 2)	Recurrent infections in 33% of patients Below-the-knee amputation ( *n* = 1) Ischemic necrosis (n = 3) Delayed wound healing ( *n* = 4) Poor cosmetic results ( *n* = 16)
Mavili et al, 1994	4	LE	Modified Charles procedure	NR	Wrapped with elastic bandages	No progression of disease	Hypertrophic scarring ( *n* = 2)
Dumanian et al, 1996	1	LE	Charles procedure	NR	Gauze dressing	Near normal contour and appearance No spontaneous cellulitis	Skin graft loss ( *n* = 1)
Fraga et al, 2004	1	UE	Disarticulation	NR	NR	Limb disarticulation	NR
Hosnuter et al, 2006	1	LE	Limited Charles procedure—FTSG Sistrunk procedure 1 year later	NR	Physical therapy	After the second operation, the left calf measurement decreased from 106 to 57 cm	No major complications
van der Walt et al, 2009	8	LE	Delayed modified Charles procedure (negative pressure 90 mm Hg: 7 d)	NR	NR	The mean weight of lymphedematous tissue removed was 8.5 kg (range, 5–14.6 kg). A 45% improvement of the LE Functional Scale	Minor additional grafting ( *n* = 3) Transfusion of blood units ( *n* = 8) Wound breakdown ( *n* = 2)
Karonidis et al, 2010	8	LE	Charles procedure with preservation of toes	Homan's procedure—thigh	Nonadherent dressings and leg elevation	The average size reduction was of 28.75% (range, 22–37%)	NR
Pereira et al, 2010	2	LE	Tissue resection	NR	Manual lymph drainage and mechanical lymph drainage	The size of the limbs can be maintained within the normal range by following the treatment guidelines	NR
Robertson et al, 2020	2	LE	Modified Charles procedure	Preoperative decongestive therapy	Physical therapy	Improved QoL	Focal wound tenderness ( *n* = 1) Minor skin graft loss ( *n* = 1)
Damstra et al, 2020	28	LE	Shaving procedure	Preoperative short-stretch compression bandaging Circumferential SAL	Analgesic, silicone wound dressings and compression bandages	Decreased episodes of erysipelas: preoperative 17.6, postoperative 0.6	NR

Abbreviations: FTSG, full-thickness skin graft; LE, lower extremity; mm Hg, millimeters of Mercury; NR, not reported; QoL, quality of life; SAL, suction-assisted lipectomy; STSG, split-thickness skin graft; UE, upper extremity.

### Excisional Procedures

We found 15 studies reporting outcomes of excisional procedures for primary lymphedema of the extremities in 124 patients. An overview of the results is displayed in Table 4 . Studies reporting the stage of lymphedema included patients with stage III ISL or were referred to as “advanced” disease. Several excisional procedures were reported including a two-stage modified Kondoleon–Sistrunk procedure ( *n* = 2); skin-sparing subcutaneous tissue excision ( *n* = 11); the Charles' procedure ( *n* = 16), the modified Charles ( *n* = 6), and delayed modified Charles ( *n* = 8); the standard Homan's procedure ( *n* = 7); a single-stage ( *n* = 26), double-stage ( *n* = 10), and triple-stage modified Homan's procedure ( *n* = 2); limb disarticulation ( *n* = 1); tissue resection or shaving procedures ( *n* = 28). Most studies reported a remarkable reduction in the size of the LE, improvement of symptoms, and a reduction in the episodes of lymphangitis and cellulitis over a follow-up period ranging from 1 to 60 months. Remarkably, van der Walt et al used a modified Charles' procedure delaying skin grafting by 5 to 7 days using negative pressure dressings. An average resection of 8.5 kg of lymphedematous tissue was reported without any major complication. 48 Karonidis et al reported a modified Charles procedure with excision of the soft tissue at the dorsum of the toes while preserving the extensor tendon and its paratenon and the skin flaps at the web spaces. 49 Additionally, wedge resection was performed over the lateral and medial aspect thigh as a Homan's procedure, providing a smooth transition between the leg and the thigh. 49 In that series, 18 of 20 patients achieved satisfactory aesthetic and functional results and no recurrent infections had been reported during a 3-year follow-up. 49 Poor cosmetic results were commonly reported ( *n* = 16). The overall complication rate was 46%; these included injury of the internal saphenous nerve ( *n* = 1), blood loss requiring transfusion ( *n* = 13), delayed wound healing ( *n* = 11), dermatosis ( *n* = 1), skin graft loss ( *n* = 6), presence of crevices and pits ( *n* = 1), chronic ulceration ( *n* = 1), the need of scar revision and release ( *n* = 2), reintervention ( *n* = 1), seroma ( *n* = 1), amputation ( *n* = 2), skin necrosis ( *n* = 3), hypertrophic scarring ( *n* = 2), and focal wound tenderness ( *n* = 1).

## Discussion

The present study aimed to report on surgical treatments in the context of primary lymphedema.

Age of onset is undoubtedly relevant to the description and presentation of symptoms as well as the overall prognosis for every patient. The average age in our review was 36 years, seemingly old for most patients with primary lymphedema; this is due to the adulthood onset of the disease, as well as delays in the diagnosis. Ergo, primary lymphedema is not a synonym for childhood lymphedema.

Traditionally, primary lymphedema has been divided into categories based on the age of onset: congenital, praecox, or *tarda* , which failed to separate patients according to developmental age. To avoid miscommunication, a clearer classification has been proposed: infancy (between birth and 1 year of age), childhood (female patients between 1 and 8 years, male patients between 1 and 9 years), adolescence (female patients between 9 and 12 years, male patients between 10 and 21 years), and adulthood lymphedema (21 years or more). 85 The availability of a precise nomenclature may be helpful to successfully detect new and existing cases, with a classification based on a developmental approach.

Some considerations can be highlighted: despite the presence of diseased lymphatic structures, most patients remain at clinical stages I and II due to a probable intrinsic compensatory mechanism that stabilizes the lymphatic anomaly when conservative measures have been implemented. 86 Consequently, patients with an early diagnosis despite an abnormal lymphatic, yet balanced, function may have a better prognosis than those with long-standing untreated lymphedema. 87

On this matter, treatment for lymphedema seeks to improve symptoms, cellulitis episodes, and QoL. It is known that the mainstay treatment for lymphedema is compression therapy, which promotes mobilization of lymph to proximal areas, reduces capillary filtration, avoids tissue inflammation, and consequently reduces fat deposits and secondary fibrosis. 17 Surgical interventions in this review were synthesized into physiological procedures (LVA and VLNT) and volume reduction or excisional surgeries (SAL and excisional procedures).

Although a clear-cut for determining the required treatment based on the severity stage could be desired, this is not that straightforward. Hence, physiological procedures should be contemplated even if a patient responds well to compression alone: a next-to-normal extremity after a physiological surgery can enable a patient to discontinue the use of a compressive garment, with the accompanying improvement in QoL. 22 Many patients may require more active compression with pneumatic devices, but these were not mentioned explicitly in the reviewed reports.

Despite an absence of uniformity in the reported surgical outcomes, circumferential measurements for volume reduction, episodes of cellulitis, improvement of symptoms, and QoL assessments were somewhat commonly evaluated. Hopefully, lymphedema guidelines should develop a standard method for expressing outcome measures.

LVA was overall the most performed procedure in this review. The size reduction of the affected limbs observed after this procedure in the studies of primary lymphedema patients is remarkable. Of note, isolated reports showed that LVA conditioned an increase in circumference in some patients, 15 53 especially those with an earlier onset of the disease. 15 Higher circumference reduction rates were observed for LVA procedures compared to VLNT, although this should be considered with caution since the sample sizes were heterogeneous. Nevertheless, from our perspective, LVA and VLNT may be considered equivalent in this respect. Finally, both LVA and VLNT improved symptoms and decreased cellulitis episodes. The complication rates appear to be higher in VLNT compared to LVA, owing to the higher complexity of the former. However, for both groups, only some complications were reported.

Since an intrinsic subnormal lymphatic anatomy is present, an essential aspect when selecting the optimal microsurgical treatment for primary lymphedema is the preoperative morphology determination in concordance with the severity of the disease. Cheng and Liu suggest performing LVA in patients with Cheng's Lymphedema Grade 0 to early Grade 2, limb circumferential difference less than 20%, short duration of symptoms, patent lymphatic ducts on indocyanine green lymphography, and partial obstruction on Tc-99 lymphoscintigraphy. 22 For patients with a greater circumferential difference, symptoms over 5 years, and absence of patent ducts or total obstruction by imaging, VLNT should be considered. This rationale indicates that performing LVA on incompetent lymphatic vessels may not only be futile but might aggravate the clinical stage of lymphedema. Similarly, in the presence of competent lymphatic vessels, performing VLNT as a first surgical instance precludes taking advantage of the existing function through the less invasive LVA.

SAL is currently the debulking procedure of choice for lymphedema and is indicated mainly for the advanced stages of the disease. In our review, patients showed a considerable decrease in circumference and improvement in cellulitis episodes and QoL with an approximate complication rate of 14.7%. The role of postoperative compression therapy was emphasized. Additionally, SAL has shown satisfactory results when combined with physiologic procedures, as liposuction addresses the deposits of fibroadipose tissue, while LVA or VLNT corrects the lymphatic flow. 88 89 Recently, a treatment algorithm for the sequence of liposuction with LVA or VLNT for lymphedema stages II to III has been proposed. 90 Nonetheless, the outcomes of this combined treatment have not been exclusively evaluated for primary lymphedema.

Excisional procedures were usually performed in the advanced stages of lymphedema; several complications and poor cosmetic results were described. The earlier the report, the more encouraging perspective was noted, even if results were considered less than ideal.

The challenge that the treatment of primary lymphedema poses is considerable. For instance, the underdeveloped lymphatic system with either abnormal lymph vessels or lymph nodes, or even both, demands an accurate and integral delineation of the lymphatic anatomy and function before considering a physiological procedure; the altered structure and lymphangiogenesis in primary lymphedema may cause inferior surgical outcomes when compared to those obtained in secondary lymphedema. Another defiance is the scenario of bilateral primary lymphedema, where improvements in circumferential measures cannot be assessed concerning a nonaffected contralateral limb. Moreover, as some authors have considered primary lymphedema as an orphan disease, late diagnosis and delayed referral are not uncommon in these patients, which notably influence the course of the disease and treatment indications. 30 This late referral may be because most reconstructive plastic surgeons were traditionally taught that primary lymphedema was not a candidate for physiologic procedures. The reflection of this situation can be seen in the continued use of excisional procedures from its first report in 1950 to the present. Importantly, it was not possible to discern the indications for LVA, neither the preoperative planning, nor the methods of preoperative lymphatic mapping that led to such indications in each study. In this context, detailed information on imaging would be greatly useful.

Similarly, postoperative objective assessments of lymphatic function are uncommon. Furthermore, although follow-up appears to be appropriate, more than 2 years on average, we still ignore the required time of monitoring; for example, some patients may develop LVA failure due to venous reflux after 2 or 3 years. 91

To our knowledge, there are no previous systematic reviews about the whole treatment spectrum for primary lymphedema. There are two recent systematic reviews partially dealing with our subject. Tang et al focused mainly on QoL and included patients with secondary lymphedema. According to the authors, both ablative and physiologic interventions appear to provide an improvement in both generic and disease-specific quality-of-life domains, these improvements are sustained for at least 6 to 12 months postoperatively, and the choice of treatment for a particular patient is not clear, ideally determined by an experienced team on a case-by-case basis. 92 The review by Fallahian et al included 10 studies in total dealing only with lymphovenous bypass and vascularized lymph node transplant. The number of patients included was considerable ( *n* = 254); the authors claimed a statistically significant improvement in the included reports but did not support this conclusion. 93 Half of their included papers (5/10) coincide with those in our review; from our standpoint, and according to the papers we gathered, statistical significance is far from conclusive. A recent meta-analysis dealt with outcomes after microsurgical treatments for lymphedema; the results are very optimistic: patients who underwent microsurgery achieved better outcomes (limb circumference diameter reduction, reduced rates of “skin infections,” and enhanced lymphatic transport capacity). It is impossible to discern which patients and which results apply to primary lymphedema. 94

The main limitation of our study is its dependence on previous and heterogeneous studies which impacts a qualitative synthesis; for example, the scantness of studies focusing only on this pathology reflects the absence of reliable data regarding the prevalence of the disease, which to our knowledge has not been updated after 36 years. 5 Despite this, we made an effort to disaggregate the information from the included articles and analyze only and exclusively cases with primary lymphedema. About the data reviewed, the predominance of case reports, small sample case series, and lack of extensive studies dealing specifically with the surgical treatment of primary lymphedema, obstacle the categorical and unequivocal selection of treatment. In this regard, granular details that would be useful to draw conclusions are missing: number of lymphovenous anastomoses performed in each limb, objective assessment of the long-term outcomes, and number of patients with combined procedures and their outcomes, among others. Unfortunately, most of the papers deal with patient groups, outcomes, and preoperative protocols that are vastly different. Also, because different lymphedema staging methods were used in the studies reviewed, comparisons were difficult to make.

However, although only low-quality data could be drawn from existing reports, an effort was made to further clarify the current management of this condition; in addition, we must consider the ethical and methodological difficulty of designing prospective and comparative studies. Also, it is possible that a selection bias had occurred, considering that those papers with positive findings are more likely to be published, and ineffective results, especially physiologic treatment, might have not been reported and therefore not included in the analysis.

More studies focusing solely on the surgical treatment for primary lymphedema are necessary; these should include detailed preexisting lymphatic morphology through imaging, clinical and surgical specifications, homogenization, and systematization in the reporting of outcomes. In this way, the endeavor of the present work may draw attention to these issues aiding in consensus and adequate communication among different working groups. Consequently, we would recommend the use of the ISL staging system for future reports.

Notwithstanding, our review shows that some treatment can be offered: more complex and sophisticated physiological procedures for earlier presentations with more conserved microstructural anatomy. On the contrary, when the lymphatic vessels' anatomy is severely altered, fibrosis is dire, and the patient is facing the inexorable progression of the disease, excisional treatment provides some relief.

## Conclusion

Staging, clinical measurements, symptoms duration, and an accurate objective preoperative description of the lymphatic anatomy and function through imaging techniques, are central in selecting proper surgical treatment, regardless of the age of onset.

Establishing the competence of lymphatic vessels is cardinal to the selection of the ideal supermicrosurgical or microsurgical treatment or a combination of these with an excisional procedure such as suction-assisted lipectomy. To better understand surgical treatment outcomes in the future, comparative studies, hopefully randomized controlled trials, with larger samples and longer follow-ups are required.

Primary lymphedema is amenable to surgical treatment; the currently performed procedures have effectively improved symptoms and QoL in this population.

## References

[1] Grada A A Phillips T J Lymphedema: pathophysiology and clinical manifestations J Am Acad Dermatol 2017 77 06 1009 1020 29132848 10.1016/j.jaad.2017.03.022

[2] Schook C C Mulliken J B Fishman S J Grant F D Zurakowski D Greene A K Primary lymphedema: clinical features and management in 138 pediatric patients Plast Reconstr Surg 2011 127 06 2419 2431 21617474 10.1097/PRS.0b013e318213a218

[3] Greene A K Goss J A Diagnosis and staging of lymphedema Semin Plast Surg 2018 32 01 12 16 29636648 10.1055/s-0038-1635117 PMC5891654

[4] Allen E Lymphedema of the extremities. Classification, etiology and differential diagnosis: a study of three hundred cases Arch Intern Med (Chic) 1934 54 04 606 624

[5] Smeltzer D M Stickler G B Schirger A Primary lymphedema in children and adolescents: a follow-up study and review Pediatrics 1985 76 02 206 218 4022694

[6] Kinmonth J B Taylor G W Tracy G D Marsh J D Primary lymphoedema; clinical and lymphangiographic studies of a series of 107 patients in which the lower limbs were affected Br J Surg 1957 45 189 1 9 13510649 10.1002/bjs.18004518902

[7] Van Damme A Seront E Dekeuleneer V Boon L M Vikkula M New and emerging targeted therapies for vascular malformations Am J Clin Dermatol 2020 21 05 657 668 32557381 10.1007/s40257-020-00528-w

[8] Brouillard P Boon L Vikkula M Genetics of lymphatic anomalies J Clin Invest 2014 124 03 898 904 24590274 10.1172/JCI71614 PMC3938256

[9] Connell F Brice G Mortimer P Phenotypic characterization of primary lymphedema Ann N Y Acad Sci 2008 1131 140 146 18519967 10.1196/annals.1413.013

[10] Kinmonth J B Eustace P W Lymph nodes and vessels in primary lymphoedema. Their relative importance in aetiology Ann R Coll Surg Engl 1976 58 04 278 284 182058 PMC2493719

[11] Liu N F Yan Z X Wu X F Classification of lymphatic-system malformations in primary lymphoedema based on MR lymphangiography Eur J Vasc Endovasc Surg 2012 44 03 345 349 22831870 10.1016/j.ejvs.2012.06.019

[12] Wolfe J HN Kinmonth J B The prognosis of primary lymphedema of the lower limbs Arch Surg 1981 116 09 1157 1160 7283712 10.1001/archsurg.1981.01380210037007

[13] Murdaca G Cagnati P Gulli R Current views on diagnostic approach and treatment of lymphedema Am J Med 2012 125 02 134 140 22269614 10.1016/j.amjmed.2011.06.032

[14] Koshima I Inagawa K Urushibara K Moriguchi T Supermicrosurgical lymphaticovenular anastomosis for the treatment of lymphedema in the upper extremities J Reconstr Microsurg 2000 16 06 437 442 10993089 10.1055/s-2006-947150

[15] Hara H Mihara M Ohtsu H Narushima M Iida T Koshima I Indication of lymphaticovenous anastomosis for lower limb primary lymphedema Plast Reconstr Surg 2015 136 04 883 893 26086382 10.1097/PRS.0000000000001631

[16] Cheng M H Loh C YY Lin C Y Outcomes of vascularized lymph node transfer and lymphovenous anastomosis for treatment of primary lymphedema Plast Reconstr Surg Glob Open 2018 6 12 e2056 30656125 10.1097/GOX.0000000000002056 PMC6326612

[17] Mosti G Cavezzi A Compression therapy in lymphedema: between past and recent scientific data Phlebology 2019 34 08 515 522 30626269 10.1177/0268355518824524

[18] Chang D W Masia J Garza R III Skoracki R Neligan P C Lymphedema: Surgical and medical therapy Plast Reconstr Surg 2016 138(3, Suppl)209S 218S 27556764 10.1097/PRS.0000000000002683

[19] Vascular Outcomes Collaborative Desai S S Shao M Superior clinical, quality of life, functional, and health economic outcomes with pneumatic compression therapy for lymphedema Ann Vasc Surg 2020 63 298 306 31629128 10.1016/j.avsg.2019.08.091

[20] Becker C Arrive L Saaristo A Surgical treatment of congenital lymphedema Clin Plast Surg 2012 39 04 377 384 23036288 10.1016/j.cps.2012.08.001

[21] Ciudad P Manrique O J Bustos S S Comparisons in long-term clinical outcomes among patients with upper or lower extremity lymphedema treated with diverse vascularized lymph node transfer Microsurgery 2020 40 02 130 136 31489971 10.1002/micr.30508

[22] Cheng M H Liu T TF Lymphedema microsurgery improved outcomes of pediatric primary extremity lymphedema Microsurgery 2020 40 07 766 775 32652644 10.1002/micr.30622

[23] Cheng M H Chen S C Henry S L Tan B K Chia-Yu Lin M Huang J J Vascularized groin lymph node flap transfer for postmastectomy upper limb lymphedema: flap anatomy, recipient sites, and outcomes Plast Reconstr Surg 2013 131 06 1286 1298 23714790 10.1097/PRS.0b013e31828bd3b3

[24] Wilting J Becker J The lymphatic vascular system: much more than just a sewer Cell Biosci 2022 12 01 157 36109802 10.1186/s13578-022-00898-0 PMC9476376

[25] Gianesini S Rimondi E Raffetto J D Human collecting lymphatic glycocalyx identification by electron microscopy and immunohistochemistry Sci Rep 2023 13 01 3022 36810649 10.1038/s41598-023-30043-x PMC9945466

[26] Dellon A L Hoopes J E The Charles procedure for primary lymphedema. Long-term clinical results Plast Reconstr Surg 1977 60 04 589 595 333486 10.1097/00006534-197710000-00015

[27] McKEE D MEM Edgerton M T Jr The surgical treatment of lymphedema of the lower extremities Plast Reconstr Surg Transplant Bull 1959 23 05 480 492 13657722 10.1097/00006534-195905000-00002

[28] Brorson H Ohlin K Olsson G Svensson B Svensson H Controlled compression and liposuction treatment for lower extremity lymphedema Lymphology 2008 41 02 52 63 18720912

[29] Boyages J Kastanias K Koelmeyer L A Liposuction for advanced lymphedema: a multidisciplinary approach for complete reduction of arm and leg swelling Ann Surg Oncol 2015 22 03 S1263 S1270 26122375 10.1245/s10434-015-4700-3 PMC4686553

[30] French National Referral Center for Primary Lymphedema Vignes S Albuisson J Champion L Primary lymphedema French National Diagnosis and Care Protocol (PNDS; Protocole National de Diagnostic et de Soins) Orphanet J Rare Dis 2021 16 01 18 33407666 10.1186/s13023-020-01652-w PMC7789008

[31] PRISMA-P Group Shamseer L Moher D Clarke M Preferred reporting items for systematic review and meta-analysis protocols (PRISMA-P) 2015: elaboration and explanation BMJ 2015 350 g7647 25555855 10.1136/bmj.g7647

[32] PRISMA-P Group Moher D Shamseer L Clarke M Preferred reporting items for systematic review and meta-analysis protocols (PRISMA-P) 2015 statement Syst Rev 2015 4 01 1 25554246 10.1186/2046-4053-4-1 PMC4320440

[33] OCEBM Levels of Evidence Working Group The Oxford Levels of Evidence 1 2009

[34] Murad M H Sultan S Haffar S Bazerbachi F Methodological quality and synthesis of case series and case reports BMJ Evid Based Med 2018 23 02 60 63 10.1136/bmjebm-2017-110853 PMC6234235 29420178

[35] Wells G Shea B O'Connell D The Newcastle-Ottawa Scale (NOS) for assessing the quality if nonrandomized studies in meta-analyses 2012. Accessed September 1st 2023, at:http://www.ohri.ca/programs/clinical_epidemiology/oxford.asp

[36] MacKmull G Weeder S D Congenital lymphedema; case report with results of surgical correction Plast Reconstr Surg 1950 5 02 157 162 10.1097/00006534-195002000-00005 15406525

[37] Fonkalsrud E W Congenital lymphedema of the extremities in infants and children J Pediatr Surg 1969 4 02 231 236 5778343 10.1016/0022-3468(69)90397-2

[38] Tilley A R Douglas L G Staged treatment of lymphedema praecox Can Med Assoc J 1974 110 03 309 312 4590797 PMC1947277

[39] Feins N R Rubin R Crais T O'Connor J F Surgical management of thirty-nine children with lymphedema J Pediatr Surg 1977 12 03 471 476 874735 10.1016/0022-3468(77)90026-4

[40] Louton R B Terranova W A The use of suction curettage as adjunct to the management of lymphedema Ann Plast Surg 1989 22 04 354 357 2705714 10.1097/00000637-198904000-00013

[41] Dumanian G A Futrell J W Radical excision and delayed reconstruction of a lymphedematous leg with a 15 year follow-up Lymphology 1996 29 01 20 24 8721975

[42] Koshima I Nanba Y Tsutsui T Takahashi Y Itoh S Long-term follow-up after lymphaticovenular anastomosis for lymphedema in the leg J Reconstr Microsurg 2003 19 04 209 215 12858242 10.1055/s-2003-40575

[43] Fraga M FP Júnior A H Guedes Neto H J Disarticulation of the left upper extremity for treatment of giant primary lymphedema–case report Lymphology 2004 37 04 199 201 15693537

[44] Hosnuter M Buyukates M Babuccu B An unusual case of lymphedema tarda Med Sci Monit 2006 12 10 CS99 CS102 17006408

[45] Greene A K Slavin S A Borud L Treatment of lower extremity lymphedema with suction-assisted lipectomy Plast Reconstr Surg 2006 118 05 118e 121e 10.1097/01.prs.0000237020.29209.22 17016168

[46] Espinosa-de-Los-Monteros A Hinojosa C A Abarca L Iglesias M Compression therapy and liposuction of lower legs for bilateral hereditary primary lymphedema praecox J Vasc Surg 2009 49 01 222 224 19174259 10.1016/j.jvs.2008.07.073

[47] Eryilmaz T Kaya B Ozmen S Kandal S Suction-assisted lipectomy for treatment of lower-extremity lymphedema Aesthet Plast Surg 2009 33 04 671 673 10.1007/s00266-009-9351-y 19434444

[48] van der Walt J C Perks T J Zeeman B JV Bruce-Chwatt A J Graewe F R Modified Charles procedure using negative pressure dressings for primary lymphedema: a functional assessment Ann Plast Surg 2009 62 06 669 675 19461283 10.1097/SAP.0b013e318180cd24

[49] Karonidis A Chen H C Preservation of toes in advanced lymphedema: an important step in the control of infection Ann Plast Surg 2010 64 04 446 450 20224333 10.1097/SAP.0b013e3181b30416

[50] de Godoy J MP Azoubel L MO de Fátima Guerreiro Godoy M Surgical treatment of elephantiasis of the feet in congenital lymphedema to facilitate the use of a compression mechanism Int J Gen Med 2010 3 115 118 20463829 10.2147/ijgm.s8962 PMC2866551

[51] Mihara M Hayashi Y Murai N Regional diagnosis of lymphoedema and selection of sites for lymphaticovenular anastomosis using elastography Clin Radiol 2011 66 08 715 719 21524415 10.1016/j.crad.2011.03.004

[52] Yamamoto T Koshima I Yoshimatsu H Narushima M Miahara M Iida T Simultaneous multi-site lymphaticovenular anastomoses for primary lower extremity and genital lymphoedema complicated with severe lymphorrhea J Plast Reconstr Aesthet Surg 2011 64 06 812 81 21093398 10.1016/j.bjps.2010.10.011

[53] Auba C Marre D Rodríguez-Losada G Hontanilla B Lymphaticovenular anastomoses for lymphedema treatment: 18 months postoperative outcomes Microsurgery 2012 32 04 261 268 22262630 10.1002/micr.20980

[54] Suehiro K Morikage N Murakami M Yamashita O Hamano K Primary lymphedema complicated by weeping chylous vesicles in the leg and scrotum: report of a case Surg Today 2012 42 11 1100 1103 22565851 10.1007/s00595-012-0193-x

[55] Yamamoto T Yoshimatsu H Yamamoto N Narushima M Iida T Koshima I Side-to-end lymphaticovenular anastomosis through temporary lymphatic expansion PLoS ONE 2013 8 03 e59523 23536881 10.1371/journal.pone.0059523 PMC3607574

[56] Ayestaray B Bekara F π-shaped lymphaticovenular anastomosis: the venous flow sparing technique for the treatment of peripheral lymphedema J Reconstr Microsurg 2014 30 08 551 560 24683133 10.1055/s-0034-1370356

[57] Gómez Martín C Murillo C Maldonado A A Cristóbal L Fernández-Cañamaque J L Double autologous lymph node transplantation (ALNT) at the level of the knee and inguinal region for advanced lymphoedema of the lower limb (elephantiasis) J Plast Reconstr Aesthet Surg 2014 67 02 267 270 24269710 10.1016/j.bjps.2013.09.016

[58] Qiu S S Chen H Y Cheng M H Vascularized lymph node flap transfer and lymphovenous anastomosis for Klippel-Trenaunay syndrome with congenital lymphedema Plast Reconstr Surg 2014 134 01 1 5 25289360 10.1097/GOX.0000000000000099 PMC4174239

[59] Akita S Mitsukawa N Kuriyama M Comparison of vascularized supraclavicular lymph node transfer and lymphaticovenular anastomosis for advanced stage lower extremity lymphedema Ann Plast Surg 2015 74 05 573 579 25875724 10.1097/SAP.0000000000000513

[60] Ito R Wu C T Lin M CY Cheng M H Successful treatment of early-stage lower extremity lymphedema with side-to-end lymphovenous anastomosis with indocyanine green lymphography assisted Microsurgery 2016 36 04 310 315 26666982 10.1002/micr.30010

[61] Koshima I Narushima M Mihara M Lymphadiposal flaps and lymphaticovenular anastomoses for severe leg edema: functional reconstruction for lymph drainage system J Reconstr Microsurg 2016 32 01 50 55 26258914 10.1055/s-0035-1554935

[62] Chen W F Yamamoto T Fisher M Liao J Carr J The “Octopus” lymphaticovenular anastomosis: evolving beyond the standard supermicrosurgical technique J Reconstr Microsurg 2015 31 06 450 457 26086669 10.1055/s-0035-1548746

[63] Gennaro P Gabriele G Mihara M Supramicrosurgical lymphatico-venular anastomosis (LVA) in treating lymphoedema: 36-months preliminary report Eur Rev Med Pharmacol Sci 2016 20 22 4642 4653 27906440

[64] Greene A K Maclellan R A Operative treatment of lymphedema using suction-assisted lipectomy Ann Plast Surg 2016 77 03 337 340 26418771 10.1097/SAP.0000000000000597

[65] Lamprou D AA Voesten H GJ Damstra R J Wikkeling O RM Circumferential suction-assisted lipectomy in the treatment of primary and secondary end-stage lymphoedema of the leg Br J Surg 2017 104 01 84 89 27809337 10.1002/bjs.10325

[66] Lee M Perry L Granzow J Suction assisted protein lipectomy (SAPL) even for the treatment of chronic fibrotic and scarified lower extremity lymphedema Lymphology 2016 49 01 36 41 29906063

[67] Yamamoto T Yoshimatsu H Yamamoto N Complete lymph flow reconstruction: a free vascularized lymph node true perforator flap transfer with efferent lymphaticolymphatic anastomosis J Plast Reconstr Aesthet Surg 2016 69 09 1227 1233 27449876 10.1016/j.bjps.2016.06.028

[68] Chen W F Zhao H Yamamoto T Hara H Ding J Indocyanine green lymphographic evidence of surgical efficacy following microsurgical and supermicrosurgical lymphedema reconstructions J Reconstr Microsurg 2016 32 09 688 698 27487485 10.1055/s-0036-1586254

[69] Mihara M Hara H Tange S Multisite lymphaticovenular bypass using supermicrosurgery technique for lymphedema management in lower lymphedema cases Plast Reconstr Surg 2016 138 01 262 272 27348659 10.1097/PRS.0000000000002254

[70] Lee K T Park J W Mun G H Serial two-year follow-up after lymphaticovenular anastomosis for the treatment of lymphedema Microsurgery 2017 37 07 763 770 28688173 10.1002/micr.30200

[71] Stewart C J Munnoch D A Liposuction as an effective treatment for lower extremity lymphoedema: a single surgeon's experience over nine years J Plast Reconstr Aesthet Surg 2018 71 02 239 245 29246738 10.1016/j.bjps.2017.11.003

[72] Borz C Muresan M Jimborean O Modified enteromesenteric bridging operation for primary lymphedema Ann Ital Chir 2018 89 00 350 356 30337509

[73] Sachanandani N S Chu S Y Ho O A Cheong C F Lin M CY Cheng M H Lymphedema and concomitant venous comorbidity in the extremity: comprehensive evaluation, management strategy, and outcomes J Surg Oncol 2018 118 06 941 952 30261108 10.1002/jso.25237

[74] Bolletta A Di Taranto G Chen S H Surgical treatment of Milroy disease J Surg Oncol 2020 121 01 175 181 31165487 10.1002/jso.25583

[75] Giacalone G Yamamoto T Belva F The application of virtual reality for preoperative planning of lymphovenous anastomosis in a patient with a complex lymphatic malformation J Clin Med 2019 8 03 371 30884770 10.3390/jcm8030371 PMC6463145

[76] Maruccia M Pezzolla A Nacchiero E Efficacy and early results after combining laparoscopic harvest of double gastroepiploic lymph node flap and active physiotherapy for lower extremity lymphedema Microsurgery 2019 39 08 679 687 31566816 10.1002/micr.30511

[77] AlJindan F K Lin C Y Cheng M H Comparison of outcomes between side-to-end and end-to-end lymphovenous anastomoses for early-grade extremity lymphedema Plast Reconstr Surg 2019 144 02 486 496 31348365 10.1097/PRS.0000000000005870

[78] Drobot A Bez M Abu Shakra I Microsurgery for management of primary and secondary lymphedema J Vasc Surg Venous Lymphat Disord 2021 9 01 226 2330 32446874 10.1016/j.jvsv.2020.04.025

[79] Onoda S Nishimon K The utility of surgical and conservative combination therapy for advanced stage lymphedema J Vasc Surg Venous Lymphat Disord 2021 9 01 234 241 32470619 10.1016/j.jvsv.2020.05.007

[80] Scaglioni M F Meroni M Fritsche E Combining superficial and deep lymphovenous anastomosis for lymphedema treatment: preliminary results Microsurgery 2022 42 01 22 31 33394562 10.1002/micr.30701

[81] Robertson B Neville E Broering M Tobler W Recht M Muck P Multidisciplinary approach to management of severe lymphedema with one-stage radical excision and split-thickness skin grafting: report of two cases J Vasc Surg Venous Lymphat Disord 2020 8 04 658 661 32139327 10.1016/j.jvsv.2020.01.004

[82] Damstra R J Dickinson-Blok J L Voesten H GJM Shaving technique and compression therapy for Elephantiasis Nostras Verrucosa (Lymphostatic Verrucosis) of forefeet and toes in end-stage primary lymphedema: a 5 year follow-up study in 28 patients and a review of the literature J Clin Med 2020 9 10 3139 32998425 10.3390/jcm9103139 PMC7601471

[83] Hayashi A Visconti G Yang C J Hayashi N Yoshimatsu H Additional lymphaticovenular anastomosis on the posterior side for treatment of primary lower extremity lymphedema J Clin Med 2022 11 03 867 35160317 10.3390/jcm11030867 PMC8836829

[84] Mavili M E Naldoken S Safak T Modified Charles operation for primary fibrosclerotic lymphedema Lymphology 1994 27 01 14 20 8207967

[85] Greene A K Schook C C Primary lymphedema: definition of onset based on developmental age Plast Reconstr Surg 2012 129 01 221e 222e 22186578 10.1097/PRS.0b013e3182365c91

[86] Barone V Borghini A Tedone Clemente E New insights into the pathophysiology of primary and secondary lymphedema: histopathological studies on human lymphatic collecting vessels Lymphat Res Biol 2020 18 06 502 509 32716244 10.1089/lrb.2020.0037

[87] Goss J A Maclellan R A Greene A K Adult-onset primary lymphedema: a clinical-lymphoscintigraphic study of 26 patients Lymphat Res Biol 2019 17 06 620 623 30916606 10.1089/lrb.2018.0032

[88] Ciudad P Manrique O J Bustos S S Single-stage VASER-assisted liposuction and lymphatico-venous anastomoses for the treatment of extremity lymphedema: a case series and systematic review of the literature Gland Surg 2020 9 02 545 557 32420290 10.21037/gs.2020.01.13 PMC7225499

[89] Forte A J Huayllani M T Boczar D Ciudad P Manrique O Lipoaspiration and lymph node transfer for treatment of breast cancer-related lymphedema: a systematic review Cureus 2019 11 11 e6096 31723482 10.7759/cureus.6096 PMC6844538

[90] Brazio P S Nguyen D H Combined liposuction and physiologic treatment achieves durable limb volume normalization in class II-III lymphedema: a treatment algorithm to optimize outcomes Ann Plast Surg 2021 86(5S, Suppl 3)S384 S389 33976067 10.1097/SAP.0000000000002695

[91] Scaglioni M F Fontein D BY Arvanitakis M Giovanoli P Systematic review of lymphovenous anastomosis (LVA) for the treatment of lymphedema Microsurgery 2017 37 08 947 953 28972280 10.1002/micr.30246

[92] Tang N SJ Ramakrishnan A Shayan R Quality-of-life outcomes after operative management of primary and secondary lymphoedema: a systematic review ANZ J Surg 2021 91 12 2624 2636 33825306 10.1111/ans.16764

[93] Fallahian F Tadisina K K Xu K Y Efficacy of microsurgical treatment of primary lymphedema: a systematic review Ann Plast Surg 2022 88 02 195 199 34398594 10.1097/SAP.0000000000002862

[94] Kong X Du J Du X Cong X Zhao Q A meta-analysis of 37 studies on the effectiveness of microsurgical techniques for lymphedema Ann Vasc Surg 2022 86 440 4.51E8 35589027 10.1016/j.avsg.2022.04.038

