# Supplementary material for: Surgical Treatment for Primary Lymphedema: A Systematic Review of the Literature
Source: Arch Plast Surg. 2024 Apr 8;51(2):212–33. doi: 10.1055/a-2253-9859 (PMC11001464; doi:10.1055/a-2253-9859)
Supplement: Supplementary file 1 — Supplementary Material [file 10-1055-a-2253-9859-s23sep0453rev.pdf]

PRISMA 2020 Checklist

Section and Topic	Item #	Checklist item	Location where item is reported
**TITLE**			
Title	1	Identify the report as a systematic review	Title, Methods
**ABSTRACT**			
Abstract	2	See the PRISMA 2020 for Abstracts checklist	Abstract
**INTRODUCTION**			
Rationale	3	Describe the rationale for the review in the context of existing knowledge	Introduction
Objectives	4	Provide an explicit statement of the objective (s) or question(s) the review addresses	Introduction
**METHODS**			
Eligibility criteria	5	Specify the inclusion and exclusion criteria for the review and how studies were grouped for the syntheses	Methods
Information sources	6	Specify all databases, registers, websites, organisations, reference lists and other sources searched or consulted to identify studies. Specify the date when each source was last searched or consulted	Methods
Search strategy	7	Present the full search strategies for all databases, registers, and websites, including any filters and limits used	Methods (►**Fig. 1**, ►**Supplementary Table S1**)
Selection process	8	Specify the methods used to decide whether a study met the inclusion criteria of the review, including how many reviewers screened each record and each report retrieved, whether they worked independently, and if applicable, details of automation tools used in the process	Methods (►**Fig. 1**)
Data collection process	9	Specify the methods used to collect data from reports, including how many reviewers collected data from each report, whether they worked independently, any processes for obtaining or confirming data from study investigators, and if applicable, details of automation tools used in the process	Methods (►**Fig. 1)**
Data items	10a	List and define all outcomes for which data were sought. Specify whether all results that were compatible with each outcome domain in each study were sought (e.g., for all measures, time points, analyses), and if not, the methods used to decide which results to collect	Methods
	10b	List and define all other variables for which data were sought (e.g., participant and intervention characteristics, funding sources). Describe any assumptions made about any missing or unclear information	Methods
Study risk of bias assessment	11	Specify the methods used to assess risk of bias in the included studies, including details of the tool(s) used, how many reviewers assessed each study and whether they worked independently, and if applicable, details of automation tools used in the process	►**Supplementary Table S3**

(*Continued*)

 © 2024. The Author(s).

(*Continued*)

Section and Topic	Item #	Checklist item	Location where item is reported
Effect measures	12	Specify for each outcome the effect measure (s) (e.g., risk ratio, mean difference) used in the synthesis or presentation of results	–
Synthesis methods	13a	Describe the processes used to decide which studies were eligible for each synthesis (e.g., tabulating the study intervention characteristics and comparing against the planned groups for each synthesis [item #5])	Methods
	13b	Describe any methods required to prepare the data for presentation or synthesis, such as handling of missing summary statistics, or data conversions	Methods
	13c	Describe any methods used to tabulate or visually display results of individual studies and syntheses	Methods
	13d	Describe any methods used to synthesize results and provide a rationale for the choice (s). If meta-analysis was performed, describe the model(s), method(s) to identify the presence and extent of statistical heterogeneity, and software package(s) used	–
	13e	Describe any methods used to explore possible causes of heterogeneity among study results (e.g., subgroup analysis, meta-regression)	–
	13f	Describe any sensitivity analyses conducted to assess robustness of the synthesized results	–
Reporting bias assessment	14	Describe any methods used to assess risk of bias due to missing results in a synthesis (arising from reporting biases)	**►Supplementary Table S3**
Certainty assessment	15	Describe any methods used to assess certainty (or confidence) in the body of evidence for an outcome	**►Supplementary Tables S2–S4**
**RESULTS**			
Study selection	16a	Describe the results of the search and selection process, from the number of records identified in the search to the number of studies included in the review, ideally using a flow diagram	Results
	16b	Cite studies that might appear to meet the inclusion criteria, but which were excluded, and explain why they were excluded	Results (**►Fig. 1**)
Study characteristics	17	Cite each included study and present its characteristics	**►Table 1**
Risk of bias in studies	18	Present assessments of risk of bias for each included study	**►Supplementary Table S3**
Results of individual studies	19	For all outcomes, present, for each study: (a) summary statistics for each group (where appropriate) and (b) an effect estimate and its precision (e.g., confidence/credible interval), ideally using structured tables or plots	**►Supplementary Tables S1–S4**
Results of syntheses	20a	For each synthesis, briefly summarise the characteristics and risk of bias among contributing studies	**►Supplementary Tables S1–S4**

 © 2024. The Author(s).

(*Continued*)

Section and Topic	Item #	Checklist item	Location where item is reported
	20b	Present results of all statistical syntheses conducted. If meta-analysis was done, present for each the summary estimate and its precision (e.g., confidence/credible interval) and measures of statistical heterogeneity. If comparing groups, describe the direction of the effect.	–
	20c	Present results of all investigations of possible causes of heterogeneity among study results	–
	20d	Present results of all sensitivity analyses conducted to assess the robustness of the synthesized results	–
Reporting biases	21	Present assessments of risk of bias due to missing results (arising from reporting biases) for each synthesis assessed	►**Supplementary Table S2**
Certainty of evidence	22	Present assessments of certainty (or confidence) in the body of evidence for each outcome assessed	►**Supplementary Table S2**
DISCUSSION			
Discussion	23a	Provide a general interpretation of the results in the context of other evidence	Discussion
	23b	Discuss any limitations of the evidence included in the review	Discussion
	23c	Discuss any limitations of the review processes used	Discussion
	23d	Discuss implications of the results for practice, policy, and future research	Discussion
OTHER INFORMATION			
Registration and protocol	24a	Provide registration information for the review, including register name and registration number, or state that the review was not registered	–
	24b	Indicate where the review protocol can be accessed, or state that a protocol was not prepared	–
	24c	Describe and explain any amendments to information provided at registration or in the protocol	–
Support	25	Describe sources of financial or nonfinancial support for the review, and the role of the funders or sponsors in the review	Disclosures
Competing interests	26	Declare any competing interests of review authors	Disclosures
Availability of data, code, and other materials	27	Report which of the following are publicly available and where they can be found template data collection forms; data extracted from included studies; data used for all analyses; analytic code; any other materials used in the review	–

Adapted from Page MJ, McKenzie JE, Bossuyt PM, et al. The PRISMA 2020 statement: an updated guideline for reporting systematic reviews. BMJ 2021;372:n71. doi: 10.1136/bmj.n71.
For more information, visit http://www.prisma-statement.org/.

 © 2024. The Author(s).

Supplementary Material

Supplementary Table S1 Search terms and boolean operators used in the systematic review

**PubMed - MEDLINE (Inception through December 2022)**
([Lymphedema] OR [Lymphoedema]) AND ([Primary] OR [Hereditary] OR [Congenital] OR [Praecox] OR [Tarda] OR [Meige's syndrome] OR [Milroy's disease]) AND ([Lymph node transfer] OR [lymphaticovenular anastomosis] OR [Lymphovenous anastomosis] OR [Liposuction] OR [Lipectomy] OR [lymph node transplant] OR [Excision] OR [radical reduction preservation perforators]) NOT ([Conservative] OR [Compression] OR [Cancer-related] OR [Mastectomy] OR [Postmastectomy] OR [treatment-related] OR [Oncologic] OR [Breast cancer] OR [Post-breast] OR [Filarial] OR [Filariasis] OR [Animal] OR [Animals] OR [Congress] OR [Cadaver] OR [Cadavers] OR [Reply] OR [Leiomyosarcoma] OR [Vulvar Cancer] OR [nonsurgical] OR [gynecologic malignancy] OR [gynecologic malignancies] OR [melanoma] OR [Lymphadenectomy])
**Web of Science (search limit: Title, Abstract, and Keywords; January 2001 through December 2022)**
([primary] OR [congenital] OR [hereditary]) AND [Lymphedema] NOT ([Secondary Lymphedema] OR [cancer-related] OR [breast cancer) OR [Filariasis] OR [filarial] OR [Carcinoma] OR [Melanoma] OR [Mastectomy])
**SCOPUS (search limit: Title, Abstract, and Keywords; Inception through December 2022)**
(Primary AND Lymphedema) OR (Hereditary AND Lymphedema) OR (Congenital AND Lymphedema) NOT (Secondary Lymphedema)
**The Cochrane Central Register of Controlled Trials (Inception through December 2022)**
Primary Lymphedema

Supplementary Table S2 Oxford Centre for Evidence-Based Medicine: Levels of Evidence

Grade of recommendation	Therapy, Prevention, Etiology, Harm[a]
**1a**	Systematic review (with homogeneity) of randomized controlled trials
**1b**	Individual randomized controlled trial (with narrow confidence interval)
**1c**	All or none. Met when all patients died before the Rx became available, but some now survive on it; or when some patients died before the Rx became available, but none now die on it
**2a**	Systematic review (with homogeneity[b]) of cohort studies
**2b**	Individual cohort study (including low-quality randomized controlled trials)
**2c**	"Outcomes" research; Ecological studies
**3a**	Systematic review (with homogeneity[b]) of case–control studies
**3b**	Individual case–control study
**4**	Case series (and poor-quality cohort and case–control studies)
**5**	Expert opinion without explicit critical appraisal, or based on physiology, bench research, or "first principles"

[a]Oxford Centre for Evidence-Based Medicine: Levels of Evidence (March 2009).
[b]By homogeneity we mean a systematic review that is free of worrisome variations (heterogeneity) in the directions and degrees of results between individual studies.

 © 2024. The Author(s).

Supplementary Table S3 Newcastle–Ottawa quality assessment scale cohort studies

**Selection**[a]
1. Representativeness of the exposed cohort
• Truly representative of the average (described) in the community
• Somewhat representative of the average in the community
2. Selection of the nonexposed cohort
• Drawn from the same community as the exposed cohort
3. Ascertainment of exposure
• Secure record (e.g., surgical records)
• Structured interview
4. Demonstration that outcome of interest was not present at the start of study
• Yes
**Comparability**[b]
Comparability of cohorts on the basis of the design or analysis
• Study controls for _____________ (select the most important factor)
• Study controls for any additional factor (These criteria could be modified to indicate specific control for a second important factor.)
**Outcome**[a]
1. Assessment of outcome
• Independent blind assessment
• Record linkage
2. Was follow-up long enough for outcomes to occur?
• Yes (select an adequate follow up period for outcome of interest)
3. Adequacy of follow-up of cohorts
• Complete follow-up: all subjects accounted for
• Subjects lost to follow up unlikely to introduce bias: small number lost, >____% (select an adequate percentage) follow-up, or description provided of those lost

[a]A study can be awarded a maximum of one star for each numbered item within the “Selection” and “Outcome” categories.
[b]A study can be awarded a maximum of two stars can be given for “Comparability” category.

Supplementary Table S4 Tool for evaluating the methodological quality of case reports and case series

**Tool for evaluating the methodological quality of case reports and case series**[§]	
**Domains**	Leading exploratory questions
**Selection**	1. Does the patient(s) represent(s) the whole experience of the investigator (center) or is the selection method unclear to the extent that other patients with similar presentation may not have been reported?
**Ascertainment**	2. Was the exposure adequately ascertained?
	3. Was the outcome adequately ascertained?
**Causality**	4. Were other alternative causes that may explain the observation ruled out?
	5. Was there a challenge/rechallenge phenomenon?
	6. Was there a dose–response effect?
	7. Was follow-up long enough for outcomes to occur?
**Reporting**	8. Is the case(s) described with sufficient details to allow other investigators to replicate the research or to allow practitioners make inferences related to their own practice?

[§]Adapted from Murad et al.[34]

 © 2024. The Author(s).
